# Case of Empyema with Successful Operation

**Published:** 1847-06

**Authors:** B. H. Colegrove

**Affiliations:** Sardinia, Erie Co.


ART. 2. *Case of Empyema with successful operation.* By B. H. Colegrove, M. D., of Sardinia, Erie Co.

Dr. Flint:—In accordance with your request, I send you the following report of a case of Empyema, which I extract from my Note-Book.

*May* 18.—I was called to visit a little daughter of Ichabod French, in the town of Boston, aged 7 years. She had acute pneumonia, two months before, and the physician who had her in charge, informed me that he could not bleed her, or administer efficient antiphlogistic medicines as he wished to do, owing to her own perverseness, and the lack of parental authority. So the case took its own course, in no manner checked or modified by medicine or medical treatment.

She had been expected to die for a number of weeks, but as she still lingered, I was requested to see her. I called on my much esteemed friend Dr. C. Emmons, *en route*, and he accompanied me. We found the patient emaciated to a skeleton, harrassed with a most distressing dyspnœa, with intervals of a short, dry, almost brassy cough. The chin was drawn down to the right clavicle, and her respiration was like the panting of a young rabbit. The right hypochondrium much enlarged, the ribs enclosing it, elevated and separated more than on the opposite side. The whole right half of the chest dull on percussion, and auscultation gave no respiratory murmur. No discoloration of the integuments at any point, and no œdema. Respiration apparently performed by the abdominal muscles. Tongue clean, pulse 150, and feeble, articulation difficult, bowels regular, night sweats profuse.

Having a strong conviction that a large quantity of pus was deposited in the right cavity of the chest, with the concurrence of my medical advisers, which was readily obtained, I operated. A point was selected between the fifth and sixth ribs, four inches from their sternal extremities, and by an incision, an inch in extent, the costal pleura through with the point of a lancet, and instantly matter rushed out, and air rushed in and out, at each inspiration and expiration. I caught in a vessel two quarts, and closed the orifice with a large pledget of lint, and several turns of a bandage round the chest, with directions that it be removed once in 24 hours, the patient to be turned with the orifice in the most depending position, and in this way the matter drawn.

The relief was astonishing and instantaneous. The countemnance which before the operation was ghastly and leaden, now assumed an aspect of comfort, the breathing was easy, and we left the little patient in a quiet slumber.

Through the opening made in the chest in this case, I at the time, introduced a probe of the ordinary length, say six inches, and the enormous cavity previously occupied by the matter, received its entire length.

The patient entirely recovered, and now at the distance of fifteen years, is residing in this town, is married and the mother of a healthy child.

Within the last five years I have had repeated opportunities of examining the thorax of this patient. The right hypochondrium is very much diminished in size, the antero-posterior diameter greatly shortened, and the general contour of that region much distorted. It gives no resonance on percussion or auscultation, the heart pulsates to the right of the sterno-spinal diameter, the right arm is smaller and weaker than the left.

